# Western diet-induced obesity results in brain mitochondrial dysfunction in female Ossabaw swine

**DOI:** 10.3389/fnmol.2023.1320879

**Published:** 2023-12-14

**Authors:** Taylor J. Kelty, Chris L. Taylor, Nicole E. Wieschhaus, Pamela K. Thorne, Amira R. Amin, Christina M. Mueller, T. Dylan Olver, Darla L. Tharp, Craig A. Emter, Alexander W. Caulk, R. Scott Rector

**Affiliations:** ^1^Department of Biomedical Sciences, University of Missouri, Columbia, MO, United States; ^2^Department of Nutrition and Exercise Physiology, University of Missouri, Columbia, MO, United States; ^3^NextGen Precision Health, University of Missouri, Columbia, MO, United States; ^4^Department of Veterinary Biomedical Sciences, Western College of Veterinary Medicine, University of Saskatchewan, Saskatoon, SK, Canada; ^5^Medtronic PLC, North Haven, CT, United States; ^6^Research Service, Harry S. Truman Memorial Veterans’ Hospital, Columbia, MO, United States; ^7^Division of Gastroenterology and Hepatology, Department of Medicine, University of Missouri, Columbia, MO, United States

**Keywords:** obesity, mitochondrial dysfunction, Complex I syndrome, prefrontal cortex, hippocampus, Western diet, swine

## Abstract

Diet-induced obesity is implicated in the development of a variety of neurodegenerative disorders. Concurrently, the loss of mitochondrial Complex I protein or function is emerging as a key phenotype across an array of neurodegenerative disorders. Therefore, the objective of this study was to determine if Western diet (WD) feeding in swine [carbohydrate = 40.8% kCal (17.8% of total calories from high fructose corn syrup), protein = 16.2% kcal, fat = 42.9% kCal, and 2% cholesterol] would result in Complex I syndrome pathology. To characterize the effects of WD-induced obesity on brain mitochondria in swine, high resolution respirometry measurements from isolated brain mitochondria, oxidative phosphorylation Complex expression, and indices of oxidative stress and mitochondrial biogenesis were assessed in female Ossabaw swine fed a WD for 6-months. In line with Complex I syndrome, WD feeding severely reduced State 3 Complex I, State 3 Complex I and II, and uncoupled mitochondrial respiration in the hippocampus and prefrontal cortex (PFC). State 3 Complex I mitochondrial respiration in the PFC inversely correlated with serum total cholesterol. WD feeding also significantly reduced protein expression of oxidative phosphorylation Complexes I–V in the PFC. WD feeding significantly increased markers of antioxidant defense and mitochondrial biogenesis in the hippocampi and PFC. These data suggest WD-induced obesity may contribute to Complex I syndrome pathology by increasing oxidative stress, decreasing oxidative phosphorylation Complex protein expression, and reducing brain mitochondrial respiration. Furthermore, these findings provide mechanistic insight into the clinical link between obesity and mitochondrial Complex I related neurodegenerative disorders.

## Introduction

Lack of mitochondrial Complex I protein or function, also known as Complex I syndrome, is a key feature commonly observed in neurodegenerative disorders (Schapira et al., 1990; Navarro et al., 2009; Adav et al., 2019; Holper et al., 2019). The cause of Complex I dysfunction remains unknown; however, epidemiological evidence indicates obesity may serve a role (Picone et al., 2020; Park et al., 2022). Using a mouse model, Cavaliere et al. (2019) demonstrated diet-induced obesity causes brain mitochondrial dysfunction, possibly related to dyslipidemia and increased oxidative stress. Complex I is highly susceptible to oxidative stress due to its close proximity to electron leakage and subsequent generation of reactive oxygen species (ROS) (Deshpande and Mohiuddin, 2023). Moreover, the Complex I structure contains iron-sulfur (Fe-S) clusters that are sensitive to oxidative stress that results in inactivation and initiates a cascade of decreased respiration states further along the electron transport chain (ETC) (Read et al., 2021). Thus, it is plausible obesity-related oxidative stress contributes to Complex I syndrome in neurodegenerative disorders.

Due to a lack of human necropsy tissue and experimental controls, forming clinical links between diet-induced obesity, oxidative stress, and mitochondrial Complex I dysfunction is extremely difficult. A meta-analysis of human trials revealed WD consumption detrimentally impacts hippocampal function, but the cellular processes have yet to be elucidated (Taylor et al., 2021). Accordingly, translational neuroscience using relevant animal models is critical for advancing the molecular understanding of the etiology of disease (Eaton and Wishart, 2017; Al Dahhan et al., 2019). Swine represent an ideal preclinical model for studying neurodegenerative disorders due to the functional and structural similarities between human and swine brains (they are gyrencephalic, contain >60% white matter, and have similar developmental peaks) (Lind et al., 2007; Swindle et al., 2012; Perleberg et al., 2018; Hoffe and Holahan, 2019). Previously, using an Ossabaw swine model, our group has shown that diet-induced obesity is associated with brain insulin resistance, evidenced by decreased insulin-stimulated Akt signaling in prefrontal cortex (PFC) of pigs fed a Western diet (WD) (Olver et al., 2018). Additionally, we demonstrated that diet-induced obesity combined with pressure-overload heart failure increased indices of amyloidogenic signaling and neuroinflammation in both the PFC and hippocampi (Baranowski et al., 2021). To our knowledge, studies assessing the effects of WD-induced obesity on indices of oxidative stress and mitochondrial function in a translational large animal model have yet to be performed. Here, we address this gap by examining the hypothesis that WD-induced obesity increases indices of oxidative stress and impairs mitochondrial function in Ossabaw swine hippocampi and PFC.

## Materials and methods

### Animals and diets

Non-blinded, non-randomized, age matched female Ossabaw swine purchased between 3 and 6 months of age (Corvus Biomedical), began either a low-fat control diet (CD) (LabDiet, #5L80, St. Louis, MO, USA) (CD, *n* = 7) or WD (LabDiet, #5B4L, St. Louis, MO, USA) (WD, *n* = 4) within the first week of arriving (one WD swine brain was unable to be taken due to feasibility issues). Swine were singly housed and maintained a standard 12:12 light dark cycle at 23°C. The low-fat CD contained 18.5% kCal from protein, 10.5% kCal from fat, and 71.0% kCal from carbohydrates. WD contained carbohydrate = 40.8% kCal (17.8% of total calories from high fructose corn syrup), protein = 16.2% kcal, fat = 42.9% kCal, and 2% cholesterol. CD swine were fed 500 g/day and WD swine were fed approximately 1,000 g/day for 6 months, like our previous studies (Baranowski et al., 2021). On the day of euthansia, all swine were sedated using Telazol/xylazine 100 mg/ml respectively, dosed at 4 mg/kg Telazol and 2 mg/kg xylazine, both intramuscular. A propofol (10 mg/ml) bolus up to 6 mg/kg was administered IV for anesthesia induction. Animals were ventilated at a rate of 8–12 breaths per minute with a tidal volume of 10–20 ml/kg at 20–25 cm H_2_O pressure. Constant rate infusion (CRI) propofol was administered at 2–10 ml/kg/h intravenous determined by palpebral response and/or pedal withdrawal reflex. The brain was extracted following sedation and humane euthanasia. The dorsal hippocampus (DHpc) and PFC were dissected for experimentation. All animal protocols were approved by University of Missouri Animal Care and Use Committee prior to the start of the study.

### Serum measurements

Blood for serum analyses was collected at the time of euthanasia via the jugular vein. Serum insulin, high-density lipoprotein (HDL) cholesterol, low-density lipoprotein (LDL) cholesterol, and total cholesterol were measured through a third-party facility (IDEXX Bioanalytics). Serum glucose (Thermo Fisher, Cat#TR15221), triglycerides (Sigma-Aldrich,Cat#F6428 and Cat#T2449), and non-esterified fatty acids (Wako Chemicals, Cat#NC9567459, Cat#NC9567460, Cat#NC9567461, and Cat#NC9567464) were measured with commercially available kits. Homeostatic Model Assessment for Insulin Resistance (HOMA-IR) was calculated according to the formula: insulin (microliter unit/ml) × fasting glucose (mg/dl) / 405.

### Mitochondrial isolation and function analysis

**FIGURE 1:**
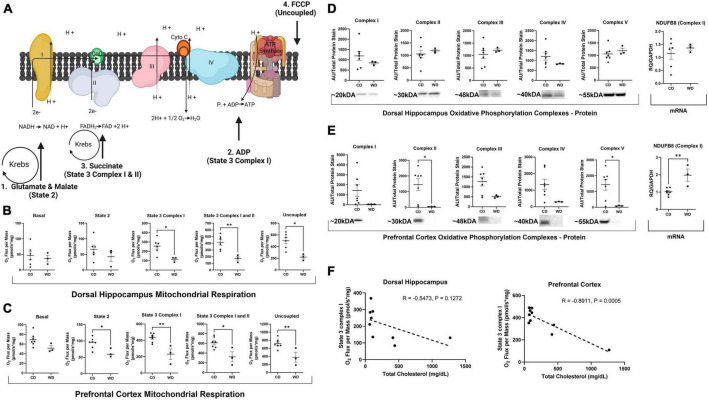
Western diet feeding-induced obesity impairs mitochondrial respiration in the brain. **(A)** Diagram representing addition of substrates to activate specific Complexes and States of respiration. 1. State 2: addition of malate and glutamate increases reduction of NAD+ into NADH by α-ketoglutarate dehydrogenase and malate dehydrogenase in the Krebs cycle. NADH then feeds into Complex I to provide a measurement of Complex I function. 2. State 3 Complex I respiration: addition of ADP (malate and glutamate already added) to stimulate electron pull through the ATPsynthase. 3. State 3 Complex I and II: addition of succinate increases reduction of FAD+ to FADH_2_ by succinic dehydrogenase in the Krebs cycle that feed FADH_2_ to Complex II. 4. Uncoupled respiration: addition of FCCP that uncouples respiration from ATPsynthase. **(B,C)** Mitochondrial respiration measured by oxygen flux without additional substrates (basal), the addition of malate and glutamate (State 2), addition of ADP (State 3 Complex I), addition of succinate (State 3 Complex I and II), and addition of FCCP (uncoupled). **(D,E)** Oxidative phosphorylation Complexes protein and transcript expression. **(F)** Correlation matrix between State 3 Complex I respiration and total serum cholesterol. “*” Indicates significant differences between groups (*p* ≤ 0.05), “**” indicates significant differences between groups (*p* ≤ 0.01). Values are presented as mean ± SE, *n* = 3–7. Panel **(A)** created with Biorender.com (2023).

As previously described (Sheldon et al., 2019; Kerr et al., 2023), DHpc and PFC were placed in mitochondria isolation buffer, homogenized with a Teflon pestle, and isolated using a percoll gradient and centrifugation. Mitochondrial respiration was assessed using high-resolution respirometry (Oroboros Oxygraph-2k; Oroboros Instruments). Basal respiration was assessed by first loading mitochondria without the addition of substrates. State 2 respiration was stimulated by the addition of malate (2 mM) and glutamate (5 mM), State 3 Complex I by titrated ADP (250–1,000 μM ADP), State 3 Complex I and II by succinate (10 mM), and maximally uncoupled with the addition of Carbonyl cyanide-p-trifluoromethoxyphenylhydrazone (FCCP) (0.25 μM). Mitochondrial respiration was normalized to protein concentration obtained from a BCA assay kit (Thermo Fisher Scientific, Cat#23227) per manufacturer’s instructions (Figure 1A).

### Western blotting

As previously described (Moore et al., 2021), 20 μg of protein lysate from whole tissue samples or precision plus protein standard (Bio-Rad, Cat#1610374) were loaded in a criterion gel, separated, transferred, blocked, and washed. Blots were imaged using SuperSignal™ West Femto Maximum Sensitivity Substrate (Thermo Fisher Scientific, Cat#34096). Protein loading was normalized by amido black staining (Sigma-Aldrich, Cat#100563). Amido black staining band densitometry was quantified using ChemiDoc XRS+ System (Bio-Rad, Model no. Universal Hood II). Primary antibodies included: Catalase (Cell Signaling, Cat#14097S), Total OXPHOS Rodent WB Antibody Cocktail (Abcam, Cat#ab110413), SOD1 (Cell Signaling, Cat#37385S), and SOD2 (Cell Signaling, Cat#13194S).

### RNA isolation, cDNA syntheses, and qRT-PCR

As previously described (Moore et al., 2021), RNA was extracted from whole DHpc and PFC tissue samples using an RNeasy kit per manufacturer’s instructions (Qiagen, Cat#74104) and a cDNA library was synthesized (Promega, Cat#A3802). A nanodrop spectrometer was used to assess RNA and cDNA purity. Quantitative real-time PCR (qRT-PCR) was performed in triplicate using SYBR Green reagents (Bio-Rad Laboratories, Cat#172–5121) and constructed primer pairs. Data are normalized to glyceraldehyde 3-phosphate dehydrogenase (GAPDH) using the 2^–ΔΔCT^ method. Forward and reverse primers are listed in Table 1.

**TABLE 1:** Forward and reverse primer sequences.

PCR primer	Forward sequence	Reverse sequence
GAPDH	5′-TCGGAGTGAA CGGATTTG	5′-CCTGGAAGATG GTGATGG
PGC1-α	5′-GATGTGTCGCC TTCTTGTTC	5′-CATCCTTTGGGG TCTTTGAG
TFAM	5′-GCTCTCCGTT CAGTTTTGCG	5′-TGCATCTGGGTT CTGAGCTTT
NDUFB8	5′-GGGGTGAACC GATACACTGG	5′-CGAAACCGGAGA GGTGCTTA
Catalase	5′-TGCAACGTTCTG TAAGGCTAGT	5′-TGCTCCTTCCAATG CTTCATCT
SOD1	5′-GTGCAGGGCACC ATCTACTT	5′-CTGCACTGGTACA GCCTTGT
SOD2	5′-GCGATCACCATG TTGTGCAG	5′-CCATAGTCGTACG GCAGGTC

### Citrate synthase activity

Citrate synthase activity was determined by spectrophotometric detection of reduced 5,5′-dithiobis (2-nitrobenzoic acid) (DTNP) in DHpc and PFC brain tissue after they were incubated in the presence of oxaloacetate and acetyl-CoA as previously described in our lab (Rector et al., 2008).

### Statistical analysis

Where possible, individual data are presented and mean data are expressed as means ± standard errors (SE). Significant differences between CD and WD were determined by an unpaired two-tailed Student’s *T*-test (*p* ≤ 0.05). Pearson correlation coefficient was computed to assess linear relationships between mitochondrial respiration and total serum cholesterol. Data were normally distributed as determined by the Shapiro–Wilk test. Effect-size correlation was calculated (*r*_Υλ_ = *d*/[(*d*^2^ + 4)^–2^] using Cohen’s *d* (*d* = *M*_1_ − *M*_2_ / σ_*pooled*_). Statistical analyses were performed with GraphPad Prism version 10.0 (Prism). No samples were excluded based on our *a priori* exclusion criteria set at 2 standard deviations from the mean; however, we were unable to obtain a sufficient hippocampal mitochondrial yield from one of the animals in the CD group.

## Results

### Animal characteristics

WD feeding significantly increased terminal body mass [*t*(9) = 8.889, *p* ≤ 0.001; Table 2], elevated serum glucose levels [*t*(9) = 3.208, *p* ≤ 0.0107; Table 2], increased HDL cholesterol [*t*(9) = 3.854, *p* = 0.0039; Table 2], increased LDL cholesterol [*t*(9) = 3.987, *p* = 0.0032; Table 2], and increased total cholesterol [*t*(9) = 4.409, *p* ≤ 0.0017; Table 2]. It is also important to note that glucose, HOMA-IR, and HDL cholesterol exceeded the desired clinical (human) range in the control group and more so in the WD fed group (Table 2). In addition, LDL and total cholesterol greatly exceeded the desired clinical (human) range in the WD fed group (Table 2).

**TABLE 2:** Animal characteristics.

Variable	CD	WD	Clinical range
Terminal body mass (Kg)	37 ± 2	65 ± 1***	N/A
Insulin (uIU/ml)	4.5 ± 0.6	5.8 ± 1.3	2.6–24.9
Glucose (mg/dl)	111 ± 6	146 ± 9*	70–105
HOMA-IR	1.3 ± 0.2	2.2 ± 0.6	<1
Triglycerides (mg/dl)	16.3 ± 0.1	23.1 ± 7.6	≤150
Non-esterified fatty acid (mEq/L)	0.4 ± 0.1	0.4 ± 0.2	N/A
HDL cholesterol (mg/dl)	46 ± 4	85 ± 12**	≥40
LDL cholesterol (mg/dl)	36 ± 4	733 ± 242**	≤130
Total cholesterol (mg/dl)	86 ± 7	824 ± 231**	150–199

Values are presented as mean ± SE. *n* = 4–7.

“*” Indicates significant differences between groups (*p* ≤ 0.05),

“**” indicates significant differences between groups (*p* ≤ 0.01),

“***” indicates significant differences between groups (*p* ≤ 0.001). “N/A” used where clinically desired range values are not applicable to swine measurements. HOMA-IR, Homeostatic Model Assessment for Insulin Resistance(; HDL, high-density lipoprotein; LDL, low-density lipoprotein.

### WD feeding decreased brain mitochondrial respiration and oxidative phosphorylation Complexes

Western diet feeding significantly decreased State 3 Complex I [*t*(7) = 2.926, *p* = 0.0221; Figure 1B] by 55%, State 3 Complex I and II [*t*(7) = 3.643, *p* = 0.0083; Figure 1B] by 59%, and uncoupled mitochondrial respiration [*t*(7) = 3.424, *p* ≤ 0.0111; Figure 1B] by 56% in the DHpc. The effect size was medium to large, ranging from 0.77 to 0.82 depending on respiration State in the DHpc. WD feeding also significantly reduced State 2 [*t*(8) = 2.816, *p* = 0.0226; Figure 1C], State 3 Complex I [*t*(8) = 4.025, *p* = 0.0038; Figure 1C] by 32%, State 3 Complex I and II [*t*(8) = 3.150, *p* = 0.0136; Figure 1C] by 47%, and uncoupled mitochondrial respiration [*t*(8) = 3.363, *p* = 0.0099; Figure 1C] by 47% in the PFC. The effect size was medium, ranging from 0.69 to 0.75 depending on respiration State in the PFC. WD did not significantly alter oxidative phosphorylation complexes in the DHpc (Figure 1D). In line with Complex I syndrome, WD feeding severely reduced oxidative phosphorylation Complexes I–V by up to 97% in the PFC (Figure 1E). Despite the severely reduced protein Complexes, WD feeding only significantly decreased Complex II [*t*(8) = 2.337, *p* = 0.0476; Figure 1E] and Complex V [*t*(8) = 2.464, *p* = 0.0391; Figure 1E] protein expression in the PFC. Additionally, NDUFB8, the transcript that encodes Complex I, was significantly elevated by WD feeding [*t*(8) = 3.933, *p* = 0.0043; Figure 1E]. A significant inverse correlation between State 3 Complex I respiration and total serum cholesterol was also detected in the PFC (*R* = −0.8911, *p* = 0.0005; Figure 1F].

### WD feeding increased antioxidant defense enzyme expression

**FIGURE 2:**
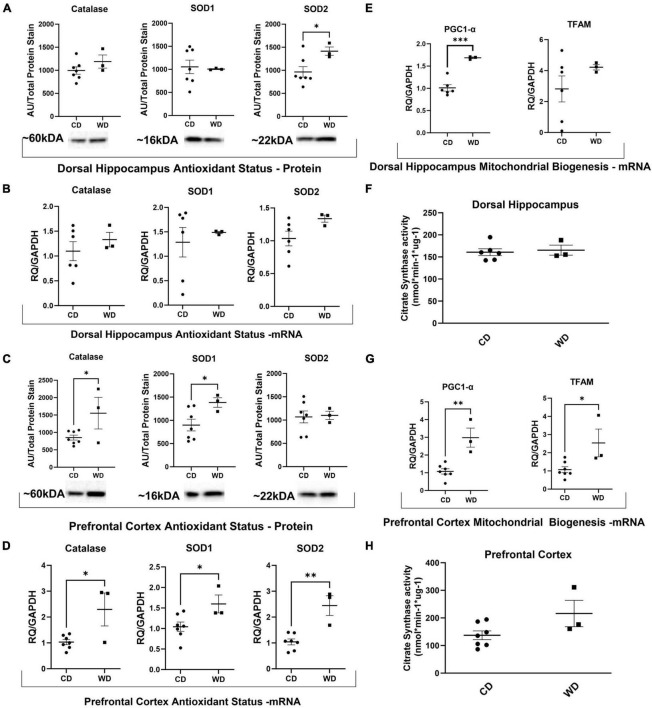
Western diet feeding-induced obesity increased markers of antioxidant status and mitochondrial biogenesis. **(A–D)** Protein and transcriptional markers of antioxidant status. **(E,G)** Transcriptional markers of mitochondrial biogenesis. **(F,H)** Citrate synthase values for all brain regions. “*” Indicates significant differences between groups (*p* ≤ 0.05), “**” indicates significant differences between groups (*p* ≤ 0.01), “***” indicates significant differences between groups (*p* ≤ 0.001). Values are presented as mean ± SE, *n* = 3–7.

Western diet feeding significantly increased SOD2 protein expression in the DHpc [*t*(8) = 2.402, *p* = 0.043; Figure 2A], but did not alter mRNA expression (Figure 2B). Furthermore, WD feeding significantly increased protein expression of catalase [*t*(8) = 2.396, *p* = 0.0435; Figure 2C] and SOD1 [*t*(8) = 2.337, *p* = 0.0476; Figure 2C] and mRNA expression of catalase [*t*(8) = 3.066, *p* = 0.0154; Figure 2D], SOD1 [*t*(8) = 2.504, *p* = 0.0367; Figure 2D], and SOD2 [*t*(8) = 4.825, *p* = 0.0013; Figure 2D] in the PFC.

### WD feeding increased transcriptional markers of mitochondrial biogenesis

Western diet feeding significantly elevated DHpc expression of PGC-1α [*t*(8) = 6.401, *p* = 0.0004; Figure 2E]. Additionally, WD feeding significantly elevated transcriptional markers of mitochondrial biogenesis in the PFC, including PPARG coactivator 1 alpha (PGC-1α) [*t*(8) = 4.796, *p* = 0.0014; Figure 2G] and mitochondrial transcription factor A (TFAM) [*t*(8) = 2.826, *p* = 0.0223; Figure 2G]. WD feeding did not significantly alter citrate synthase activity, a biomarker for mitochondrial mass, in any of the brain regions (Figures 2F, H).

## Discussion

The loss of mitochondrial Complex I protein or function is implicated in neurodegenerative disorders ranging from Parkinson’s to Alzheimer’s disease (Holper et al., 2019). To our knowledge, this is the first study to document that diet-induced obesity leads to functional Complex I pathology in a translational large animal model. Specifically, the data show diet-induced obesity is associated with decreased State 3 Complex I, I and II and uncoupled respiration in both the DHpc and PFC, and decreased State 2 respiration in the PFC alone. This may have been related to oxidative stress, as antioxidant defense enzymes were upregulated in the obese group. Further, although citrate synthase activity was not increased, diet-induced obesity increased expression of the mitochondrial biogenesis marker PGC1-α in both the DHpc and PFC, indicative perhaps of a compensatory response to counteract reduced mitochondrial respiration. Collectively, the data implicate diet-induced obesity as a contributing factor to mitochondrial Complex I dysfunction in the brain, and that early compensatory responses may include enhanced antioxidant defense as well as increased mitochondrial biogenesis signaling. These findings are critical, as they provide mechanistic insight into the clinical link between obesity and mitochondrial Complex I related neurodegenerative disorders (Picone et al., 2020; Park et al., 2022).

In the present study we interrogated the PFC and DHpc, two brain regions specifically involved with cognitive processing which declines in neurodegenerative diseases (Salat et al., 2001; Fanselow and Dong, 2010). Complex I function was reduced 32% in the PFC in the obese group. These data align with the Navarro et al. (2009) that reported a 38% reduction in Complex I activity in human frontal cortex of patients with Parkinson’s disease and Dementia with Lewy bodies. Due to the location of Complex I in the ETC, reduction in Complex I can cause a cascade of decreased respiration States further along the ETC (Deshpande and Mohiuddin, 2023). Indeed, we observed obesity-induced decreases in State 3 Complex I, State 3 Complex I and II, and uncoupled respiration by 45–60% in the DHpc and PFC. Further, there was a dramatic quantitative reduction in protein expression of all oxidative phosphorylation Complexes in the PFC, with Complexes II and V significantly reduced. Thus, both impaired Complex function and decreased complex expression may account for the reduced mitochondrial respiration in the PFC. Although the underlying cause of these maladaptations is likely multi-factorial, previous literature indicates diet-induced obesity can cause mitochondrial dysfunction and oxidative stress (Cavaliere et al., 2019). Supporting this prospect, obese pigs displayed increased SOD1, SOD2, and catalase at the transcript and protein level. Collectively, these enzymes are part of the antioxidant defense system involved in converting superoxide (O_2_^–^) into oxygen and water (Demirci-Çekiç et al., 2022), where upregulation of these enzymes may reflect a compensatory response to a greater ROS burden.

Despite functional impairments in mitochondrial respiration in obese pigs, citrate synthase content, a marker of mitochondrial content, was unaffected in the current study. Previously, it has been reported that Complex I pathology is associated with increased mitochondrial mass in humans (Navarro et al., 2009). Although our data do not show an increase mitochondrial content, consistent with this earlier observation, PGC1-α expression, a marker of mitochondrial biogenesis, was upregulated in both the DHpc and PFC. Concurrently, there was an increase in Complex I transcript expression in the PFC of obese pigs. Taken together, like the increase in SOD1, SOD2, and catalase, increases in PGC1-α and Complex I transcript expression may reflect early compensatory adaptations to counteract decreased Complex protein content and mitochondrial respiration. However, without longitudinal data, this prospect cannot be confirmed.

A major strength of this current study was the use of a translational large animal model, as pig brains share structural and functional similarities with humans (Hoffe and Holahan, 2019). Due to its size, the pig brain also offers the possibility to isolate large amounts of mitochondria that ensures stability of respiratory parameters when isolating from distinct brain regions (Fišar and Hroudová, 2016), which may enhance the predictive value of this preclinical model (Perleberg et al., 2018). Additionally, this study focused on females, which have greater cognitive deterioration compared to males at the same stage of neurodegenerative disease (Cerri et al., 2019; Williamson et al., 2022). However, several limitations must be considered when interpreting the findings of the present study. Namely, the study design was limited to a small sample of swine, and the WD intervention was of a relatively short duration. Nevertheless, because Ossabaw swine are also highly susceptible to diet-induced obesity and display human-like weight-related clinical comorbidities (Zhang et al., 2021), our WD feeding protocol was able to induce obesity and other weight-related clinical comorbidities like that of humans. WD feeding induced obesity was confirmed by the significantly increased terminal body mass, and we have previously demonstrated that a similar duration of WD feeding in Ossabaw swine results in significant increases in % body fat (Panasevich et al., 2018). Additionally, WD-induced obesity increased indices of insulin resistance (elevated blood glucose levels and a HOMA-IR above 2) and dyslipidemia (LDL and total cholesterol greatly exceeded the desired clinical range). In line with the obese phenotype, we observed medium to large effect sizes ranging from 0.69 to 0.82 for our primary outcome variables as well as a strong correlation between State 3 respiration and total serum cholesterol. This correlation builds on previous work that reported WD-induced increases in cholesterol are coupled with reduced mitochondrial respiration in mice (Cavaliere et al., 2019; Mancini et al., 2021), but requires clinical validation. Unfortunately, cognitive function experiments were not performed in the current study. However, other groups have demonstrated neurodegeneration without cognitive impairment after only 10 weeks of high fat diet feeding in juvenile Iberian pigs (Zeltser et al., 2020), suggesting neurodegeneration is detectable before cognitive impairment. Overall, the data implicate diet-induced obesity in the loss of mitochondrial Complex I function in the brain and highlight that several robust defense mechanisms including increased antioxidant status, increased Complex I expression, and increased mitochondrial biogenesis as potential targets for future research.

Lack of mitochondrial Complex I protein or function is a feature observed in an array of neurodegenerative disorders (Schapira et al., 1990; Navarro et al., 2009; Adav et al., 2019; Holper et al., 2019), including those associated with increased adiposity (Picone et al., 2020; Park et al., 2022). Yet, the cause of Complex I dysfunction has yet to be elucidated (Wang et al., 2022). The current study is the first to provide mechanistic insight from a translational large animal model and indicates that diet-induced obesity contributes to Complex I dysfunction in the brain. Considering this was observable in juvenile animals, that were obese for a relatively short duration, suggests this may represent an early feature in the pathophysiology of disease. More research dedicated to understanding the molecular signature of pathology and data from human necropsy tissue are needed to confirm the clinical relevance of these findings. Additionally, future studies should also include cognitive functional experiments to delineate clinical symptomology from loss of brain mitochondria respiration.

## Data availability statement

The original contributions presented in this study are included in this article/supplementary material, further inquiries can be directed to the corresponding author.

## Ethics statement

The animal study was approved by the University of Missouri Animal Care and Use Committee. The study was conducted in accordance with the local legislation and institutional requirements.

## Author contributions

TK: Formal analysis, Investigation, Methodology, Supervision, Conceptualization, Data curation, Software, Writing – original draft. CT: Data curation, Formal analysis, Methodology, Writing – review & editing. NW: Data curation, Formal analysis, Methodology, Writing – review & editing. PT: Methodology, Resources, Supervision, Writing – review & editing. AA: Methodology, Resources, Writing – review & editing. CM: Methodology, Resources, Writing – review & editing. TO: Conceptualization, Investigation, Supervision, Writing – review & editing. DT: Methodology, Resources, Supervision, Writing – review & editing. CE: Methodology, Resources, Supervision, Writing – review & editing. AC: Methodology, Resources, Writing – review & editing. RR: Formal analysis, Funding acquisition, Investigation, Methodology, Resources, Supervision, Writing – review & editing, Conceptualization.
